# Synchronization analyze of *k*-uniform hyper-networks

**DOI:** 10.1038/s41598-024-56198-9

**Published:** 2024-03-13

**Authors:** Juan Du, Xiujuan Ma, Fuxiang Ma, Wenqian Yu

**Affiliations:** 1 School of Computer Science, Qinghai Normal University, Xining, 810000 Qinghai China; 2 The State Key Laboratory of Tibetan Intelligent Information Processing and Application, Qinghai Normal University, Xining, 810016 Qinghai China

**Keywords:** Computer science, Scientific data

## Abstract

Hyper-networks tend to perform better in representing multivariate relationships among nodes. Yet, due to the complexity of the hyper-network structure, research in synchronization dynamics is rarely involved. In this paper, a Kuramoto model more suitable for *k*-uniform hyper-networks is proposed. And the generalized Laplacian matrix expression of the *k*-uniform hyper-network is present. We use the eigenvalue ratio of the generalized Laplacian matrix to quantify synchronization. And we studied the effects of some important structure parameters on the synchronization of three types of *k*-uniform hyper-networks. And obtained different relationships between synchronization and these parameters. The results show the synchronization of the *k*-uniform hyper-networks is related to both structure and parameters. And as the size of the nodes increases, the synchronization ability gradually increases for ER random hyper-network, while that gradually decreases for NW small-world hyper-network and BA scale-free hyper-network. As the uniformity increases, the synchronization ability of all three types of uniform hyper-networks increases. In addition, when the structure and node size are fixed, the synchronization ability increases with the increase of the hyper-clustering coefficient in BA scale-free hyper-network and ER random hyper-network, while it decreases with the increase of the hyper-clustering coefficient in NW small-world hyper-network.

## Introduction

With the development of information technology, complex networks have become a powerful tool for analyzing real systems. Currently, research focuses on using complex networks to model real systems^1–3^ and analyze their dynamical behaviors^4–7^. With the rapid development of human society, whether it is a single system or a system formed by many systems, the connections between nodes begin to become more complex and diverse. Previous graph-based complex networks could only represent pairwise connections between two nodes, which could not express the complex coupling relationships between many nodes^8–10^. Hypergraph-based hyper-networks provide a new research idea for the representation of complex systems with many nodes coupling. The hyper-edge in hypergraph can describe a certain complex connection by many nodes^11–14^. While the different connections between nodes within hyper-edge can also better represent the influence of microstructure on the dynamics of hyper-networks^15^.

Synchronization is a common class of nonlinear physical phenomena that exists in nature. Such as the synchronous luminescence of fireflies^16^ and the synchronous activity of cardiac muscle cells and nerve cells in the brain^17^. Complex network synchronization refers to the state of nodes in the network reaching consistency or similarity through mutual coupling, at which point the network can be considered synchronized. Synchronization is an important dynamical behavior of complex systems, the study results of which can help people better understand and explain the synchronization phenomena in the real world. Synchronization is divided into controlled synchronization^18–20^ and self-coupled synchronization^21–23^. Controlled synchronization means that the variables or systems are synchronized after added some control strategies. Self-coupled synchronization means that the variables or systems are synchronized by information exchange through coupling relationships.

The purpose of studying the synchronization of complex networks is to better understand the influence of network structure on the dynamic behaviors of complex networks and then to further improve the synchronization ability of the system according to practical needs. For instance, a localized breakdown in the power system may lead to more outages and it is necessary to reduce the occurrence of accidents by increasing frequency synchronization. With the gradual progress of the study of complex network structure, the study of the synchronization of complex networks has become increasingly mature. Yet, due to the complexity of the hyper-network structure, most studies on synchronization have focused on graph-based complex networks, while the study of hyper-network synchronization is not deep enough. Daniel Irving et al.^24^ gave a general framework for studying the synchronization stability of super-networks by performing simultaneous chunking diagonalization operations on matrices while reducing the dimensionality of the problem. Anwar et al.^25^ proved that the intra-layer synchronous state in a multilayer hyper-network is an invariant solution, obtained the condition for the stability of the coherent state under the time-averaged network structure, and finally obtained the condition for the stability of the synchronous state without considering the time-averaged network structure by diagonalizing the coupling matrix in blocks simultaneously. Sarbendu Rakshit et al.^26^ studied the inter-layer synchronization of stochastic multilayer hyper-networks under two different connectivity mechanisms and obtained invariance and stability conditions for the inter-layer synchronization manifolds. Sorrentino et al.^27^ constructed a super-network coupled by two or more different networks, obtained the approximate form of the principal stability function using the principal stability function method, and generalized the stability results to the general hyper-network case by constructing neural hyper-networks. Wu et al.^28^ introduced the joint degree to construct an evolving hyper-network model and obtained several simple and effective synchronization criteria for evolutionary hyper-networks. Meanwhile, Tang et al.^29^ considered the Kuramoto model with 2-hyperlink interactions to get the synchronization ability of undirected higher-order networks and proposed a synchronization optimization criterion and proved that a symmetric structure can maintain the best synchronization ability in directed higher-order networks. Maxime Lucas et al.^30^ proposed a general framework for the Kuramoto model under higher-order structures and introduced a multi-order Laplacian operator whose spectrum determines the stability of the simultaneous solutions.

At present, the studies on the synchronization dynamics of hyper-network are not considered the influence of hyper-network structure. However, studying the influence of the hyper-network structure on the synchronization ability will better discover the relationship between the hyper-network structure and synchronization. And provides reliable theoretical support to further understand the hyper-network synchronization behavior.

To this end, first, this paper proposes a general expression for the Kuramoto model of *k*-uniform hyper-networks. And present an expression for the generalized Laplacian matrix of *k*-uniform hyper-networks. We use the eigenvalue ratio of the generalized Laplacian matrix to quantify synchronization. Next, the synchronization abilities of the 3-uniform BA scale-free hyper-network, the 3-uniform NW small-world hyper-network, and the 3-uniform ER random hyper-network are studied in detail, using the 3-uniform hyper-network as an example. In addition, by analyzing the relationship between the hyper-clustering coefficient and the synchronization ability under different hyper-network structures, this paper obtains the hyper-clustering coefficient can be used as a measure to characterize the synchronization ability of the hyper-network.

### Related knowledge and calculation

#### Hypergraph

Let *H* = (*V*,*E*) be a hypergraph^31^, where *V* is the set of nodes,$V = \left\{ {v_{1} ,v_{2} , \ldots ,v_{N} } \right\}$; *E* is the set of hyper-edges,$E = \left\{ {e_{1} ,e_{2} , \ldots ,e_{M} } \right\}$, and $e_{i} \ne \emptyset \left( {i = 1,2, \ldots ,M} \right)$. The number of node is *N* and the number of hyperedge is *M* in the hypergraph. If node $v_{i}$ and node $v_{j}$ belong to the same hyper-edge $e_{k}$, then $v_{i} \in e_{k} \;{\text{and}}\;v_{j} \in e_{k}$. If hyper-edges $e_{i}$ and $e_{j}$ are adjacent to each other, then $e_{i} \cap e_{j} \ne \emptyset$. If all nodes inside a hyper-edge $e_{i}$ do not belong to any other hyper-edges, the hyper-edge $e_{i }$ is said to be an isolated hyper-edge.

#### Uniform hypergraph and non-uniform hypergraph

**Figure 1:**
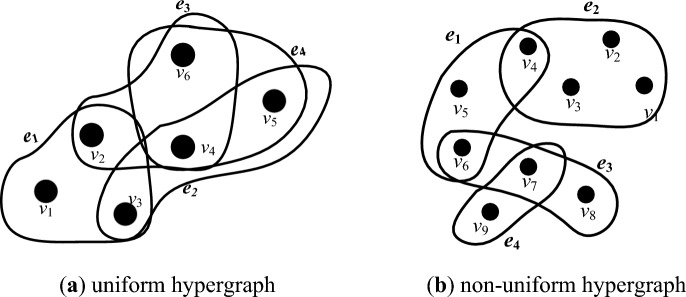
Uniform hypergraph and Non-uniform hypergraph.

Let $\left| {e_{i} } \right|$ be the number of nodes contained in the hyper-edge $e_{i}$. The rank of the hypergraph *H* is the maximum value of the number of nodes contained in the hyper-edge, denoted as $r\left( H \right) = max\left| {e_{i} } \right|,\left( {i = 1,2, \ldots , M} \right)$. The co-rank of the hyper-graph *H* is the minimum value of the number of nodes contained in the hyper-edges, denoted as $cr\left( H \right) = min\left| {e_{i} } \right|,\left( {i = 1,2, \ldots , M} \right)$. If the hypergraph *H* satisfies $r\left( H \right) = { }cr\left( H \right) = k$, the hypergraph is said to be a uniform hypergraph or a *k*-uniform hypergraph^31^. Conversely, the hypergraph *H* is said to be a non-uniform hypergraph. A hyper-network constructed based on a uniform hypergraph is a uniform hyper-network. A hyper-network constructed based on a non-uniform hypergraph is a non-uniform hyper-network. Figure 1a show a 3-uniform hypergraph with 6 nodes and 4 hyper-edges; Fig. 1b shows a non-uniform hypergraph with 9 nodes and 4 hyper-edges.

#### Hyper-adjacent matrix

Let the hyper-network *H* = (*V*,*E*) be a *k*-uniform hyper-network containing *k* nodes at each hyper-edge.$A_{{H_{k} }} = (a_{{i_{1} ,i_{2} , \ldots ,i_{k} }} )\underbrace {{_{N \times N \times \cdots \times N} }}_{k}\left( {i_{1} ,i_{2} , \ldots ,i_{k} \in \left\{ {1,2, \ldots ,N} \right\}} \right)$ is a *k*-dimensional and *N*-order symmetric matrix, that represents the hyper-adjacent matrix of the *k*-uniform hyper-network^14^. And $a_{{i_{1} ,i_{2} ,...,i_{k} }}$ denote the coupling relationship between any *k* nodes in the hyper-network, then:

1 $$ a_{{i_{1} ,i_{2} , \ldots ,i_{k} }} = \left\{ {\begin{array}{*{20}l} {1,} \hfill & {\left\{ {i_{1} ,i_{2} , \ldots ,i_{k} } \right\} \subseteq \exists e_{i} ,i = 1,2, \ldots ,M} \hfill \\ {0,} \hfill & {\left\{ {i_{1} ,i_{2} , \ldots ,i_{k} } \right\} \not\subset \forall e_{i} ,i = 1,2, \ldots ,M} \hfill \\ \end{array} } \right.. $$

#### Hyper-clustering coefficient

The clustering coefficient is used to describe the closeness between nodes in a network. In a hyper-network, the hyper-clustering coefficient of node *i* is defined as the ratio of the number of “hyper-triangles” formed by the node to the number of “2-hyper-paths” formed by the node. A hyper-triangle is a sequence of 3 different nodes and 3 different hyper-edges. And a 2-hyper-path is a sequence of 3 different nodes and 2 different hyper-edges^32^. As in Fig. 1a, *v*_6_, *e*_3_, *v*_4_, *e*_2_, *v*_5_, *e*_4_, *v*_6_ is a hyper-triangle, while *v*_5_, *e*_2_, *v*_4_, *e*_4_, *v*_4_, *e*_2_, *v*_5_ is not a hyper-triangle because there have identical nodes and hyper-edges, which is a “false hyper-triangles”; then *v*_6_, *e*_3_, *v*_2_, *e*_1_, *v*_3_ is a 2-hyper-path.

The hyper-clustering coefficient $H_{{C_{i} }}$ of node *i* is defined as:

2 $$ H_{{C_{i} }} = \frac{{6 \times N_{i\Delta } }}{{N_{i\Lambda } }} $$

where $N_{{i{\Delta }}}$ is the number of hyper-triangles formed from node *i* and $N_{{i{\Lambda }}}$ is the number of 2-hyper-paths formed from node *i*.

The average hyper-clustering coefficient *H*_*C*_ of the hyper-network is defined as:

3 $$ H_{C} = \frac{1}{N}\sum\limits_{i = 1}^{N} {H_{{C_{i} }} } $$

The hyper-clustering coefficient of the hyper-network shown in Fig. 1a is *H*_*C*_ = 1.555 and the hyper-clustering coefficient of individual nodes is shown in Table 1.

**Table 1:** Number of hyper-triangles, 2-hyper-paths and the hyper-clustering coefficient.

	*v*_1_	*v*_2_	*v*_3_	*v*_4_	*v*_5_	*v*_6_
Hyper-triangles	0	2	2	4	2	2
2-Hyper-paths	4	8	6	8	8	9
Hyper-clustering coefficient	0	1.5	2	3	1.5	1.33

#### Joint degree

Let $d_{{i_{1} i_{2 \ldots } i_{k - 1} }}$ be the joint degree of node $i_{1} i_{2 \ldots } i_{k - 1}$, which denotes the number of hyper-edges containing nodes $i_{1} i_{2 \ldots } i_{k - 1}$. Let $JD_{{H_{k} }}$ is the joint degree matrix of the hyper-network^28^, then

4 $$ JD_{{H_{k} }} = (Jd_{{i_{1} i_{2} \ldots i_{k - 1} }} )\underbrace {{_{N \times N \times \cdots \times N} }}_{k - 1} = \left\{ {\begin{array}{*{20}l} {p;} \hfill \\ {0,} \hfill \\ \end{array} } \right. $$

5 $$ Jd_{{i_{1} i_{2} \ldots i_{k - 1} }} = \sum\limits_{{i_{1} = 1}}^{N} {a_{{i_{1} i_{2} \ldots i_{j} \ldots i_{k} }} } = \sum\limits_{{i_{2} = 1}}^{N} {a_{{i_{1} i_{2} \ldots i_{j} \ldots i_{k} }} } = \cdots = \sum\limits_{{i_{j} = 1}}^{N} {a_{{i_{1} i_{2} \ldots i_{j} \ldots i_{k} }} } = \cdots = \sum\limits_{{i_{k} = 1}}^{N} {a_{{i_{1} i_{2} \ldots i_{j} \ldots i_{k} }} } . $$

The joint degree matrix of the hyper-network is shown in Fig. 1a as follows:

$$ JD_{{H_{3} }} = \left( {\begin{array}{*{20}c} {} & {v_{1} } & {v_{2} } & {v_{3} } & {v_{4} } & {v_{5} } & {v_{6} } \\ {v_{1} } & 0 & 1 & 1 & 0 & 0 & 0 \\ {v_{2} } & 1 & 0 & 1 & 1 & 0 & 1 \\ {v_{3} } & 1 & 1 & 0 & 1 & 1 & 0 \\ {v_{4} } & 0 & 1 & 1 & 0 & 2 & 2 \\ {v_{5} } & 0 & 0 & 1 & 2 & 0 & 1 \\ {v_{6} } & 0 & 1 & 0 & 2 & 1 & 0 \\ \end{array} } \right). $$

#### Kuramoto model of k-uniform hyper-network

Inspired by reference^29,30^, in this paper, we propose the Kuramoto model that is more suitable for describing *k*-uniform hyper-network synchronization, as shown in (6):

6 $$ \mathop {\theta_{{i_{j} }} }\limits^{ \cdot } = f(\omega_{{i_{j} }} ) + K\sum\limits_{{i_{1} = 1}}^{N} {\sum\limits_{{i_{2} = 1}}^{N} { \cdots \sum\limits_{{i_{j} = 1}}^{N} { \cdots \sum\limits_{{i_{k} = 1}}^{N} {{\text{a}}_{{i_{1} i_{2} \ldots i_{j} \ldots i_{k} }} g\left( {\theta_{{i_{1} }} \theta_{{i_{2} }} \ldots \theta_{{i_{j} }} \ldots \theta_{{i_{k} }} } \right)} } } } $$

where $\theta_{{i_{j} }} = [0,2\pi )$ denotes the phase of the node *i*_*j*_, *f*(·) is the local dynamical function used to describe the natural frequency of the node, *K* is the coupling constant, and *N* is the number of nodes of the hyper-network, ${\text{a}}_{{i_{1} i_{2} ...i_{j} ...i_{k} }}$ is the hyper-adjacent matrix of the *k*-uniform hyper-network, which represents the coupling relationship between any *k* nodes, if *k* nodes belong to the same hyper-edge then ${\text{a}}_{{i_{1} i_{2} ...i_{j} ...i_{k} }} = 1$, otherwise, ${\text{a}}_{{i_{1} i_{2} ...i_{j} ...i_{k} }} = 0$. *g* is the coupling function for synchronization, which is usually taken as *g* = $\sin \theta$. For instance, when the coupling occurs between two nodes $\theta_{{i_{1} }}$ and $\theta_{{i_{2} }}$, then $g(\theta_{{i_{1} }} ,\theta_{{i_{2} }} ) = \sin (\theta_{{i_{2} }} - \theta_{{i_{1} }} )$^29^.

#### Generalized Laplacian matrix of k-uniform hyper-network

When considering the synchronization between nodes of the hyper-network, the synchronization ability of the hyper-network can be obtained by the master stability equation analysis method. Since the Jacobi term is constant in the main stability equation, the stability of the main stability equation only depends on the generalized Laplacian matrix^30^, further the synchronization ability can be expressed by the eigenvalues of the generalized Laplacian matrix^29^. In the master stability equation-based analysis method, the generalized Laplacian matrix of the node is defined as follows:

7 $$ L_{{i_{1} \;i_{2} \ldots i_{j} \ldots i_{k - 1} }} = (k - 1) \cdot d_{H} (i_{j} )\delta_{{i_{1} \;i_{2} \ldots i_{j} \ldots i_{k - 1} }} - JD_{{H_{k} }} $$

8 $$ d_{H} (i_{j} ) = \left( {\sum\limits_{{i_{1} = 1}}^{N} { \ldots \sum\limits_{{i_{j - 1} = 1}}^{N} {\sum\limits_{{i_{j + 1} = 1}}^{N} { \ldots \sum\limits_{{i_{k} = 1}}^{N} {a_{{_{{i_{{_{1} }} \ldots i_{j} i_{j + 1} \ldots i_{{_{k} }} }} }} } } } } } \right)/(k - 1) $$

where *k* denotes the number of associated nodes inside the hyper-edge, i.e., the uniform number of the *k*-uniform hyper-network; $d_{H} (i_{j} )$ denotes the hyper-degree of node *i*_*j*_; $JD_{{H_{k} }}$ is the joint-degree matrix denoted by (4). The generalized Laplacian matrix of the hyper-network shown in Fig. 1a is shown below:

$$ L = \left( {\begin{array}{*{20}c} 2 & { - 1} & { - 1} & 0 & 0 & 0 \\ { - 1} & 4 & { - 1} & { - 1} & 0 & { - 1} \\ { - 1} & { - 1} & 4 & { - 1} & { - 1} & 0 \\ 0 & { - 1} & { - 1} & 6 & { - 2} & { - 2} \\ 0 & 0 & { - 1} & { - 2} & 4 & { - 1} \\ 0 & { - 1} & 0 & { - 2} & { - 1} & 4 \\ \end{array} } \right). $$

Specially, we calculate the eigenvalues of hyper-network Laplacian matrix of (7), which can be arranged as $0 = \lambda_{1} < \lambda_{2} \le \lambda_{3} \le \ldots \le \lambda_{N}$. Note that the eigenvalues are all real, as generalized Laplacians are symmetric. The smallest nonzero eigenvalue $\lambda_{2} { }$ is known as the spectral gap. It has been proved in the reference^30^ that the eigenvalue ratio $R = \frac{{\lambda_{N} }}{{\lambda_{2} }}$ quantifies synchronization ability of hyper-network. By diagonalizing the Laplacian matrix (7), we can get its eigenvalues and the eigenvalue ratio^29,30^.

### Kuramoto model and Generalized Laplacian matrix of 3-uniform hyper-network

In this paper, we study and analyze the synchronization stability of *k*-uniform hyper-networks by using 3-uniform hyper-network as an example. From Eq. (6) and Definition (7), the expression of the Kuramoto model for the 3-uniform hyper-network is shown in (9):

9 $$ \mathop {\theta_{i} }\limits^{.} = f(\omega_{i} ) + K\sum\limits_{j = 1}^{N} {\sum\limits_{k = 1}^{N} {a_{ijk} \sin (\theta_{j} + \theta_{k} - 2\theta_{i} )} } $$

A simultaneous state linearization of the state equation (9) yields^29^:

10 $$ \delta \mathop {\theta_{i} }\limits^{.} = K\sum\limits_{j = 1}^{N} {\sum\limits_{k = 1}^{N} {a_{ijk} (\delta \theta_{j} + \delta \theta_{k} - 2\delta \theta_{i} )} } $$

The simultaneous state stability of the linearization equation (10) can be determined by its generalized Laplacian matrix (11):

11 $$ L_{ij} = 2 \cdot d_{H} (i)\delta_{ij} - JD_{{H_{3} }} $$

12 $$ d_{H} (i) = \sum\limits_{j = 1}^{N} {\sum\limits_{k = 1}^{N} {a_{ijk} } } /2 $$

13 $$ JD_{{H_{3} }} = Jd_{{H_{3} }} (ij) = (Jd_{ijk} )_{N \times N} $$

Let the eigenvalues of the generalized Laplacian matrix of the 3-uniform hyper-network be $0 = \lambda_{1} < \lambda_{2} \le \lambda_{3} \le \cdots \le \lambda_{N}$. According to the theory of principal stability functions the synchronization ability of the 3-uniform hyper-network is determined by the minimum nonzero eigenvalue $\lambda_{2}$ or eigenvalue ratio $R = \frac{{\lambda_{N} }}{{\lambda_{2} }}$ of the generalized Laplacian matrix^30^. Generally, the smaller the eigenvalue ratio *R* or the larger the $\lambda_{2}$, the stronger the synchronization ability of the hyper-network; the larger the eigenvalue ratio *R* or the smaller the $\lambda_{2}$, the weaker the synchronization ability of the hyper-network^29^. In this paper, the eigenvalue ratio *R* is used as the basis for judging the synchronization ability of the *k*-uniform hyper-network.

#### Theoretical valuation of hyper-clustering coefficient

In this paper, we give the equation (14) for the valuation of the number of hyper-triangles and the equation (15) for the valuation of the number of 2-hyper-paths, and explore the relationship between the synchronization ability and hyper-clustering coefficient of the hyper-network.

14 $$ N_{hyper\_triangle} = d_{H} (i)\left\{ {\sum\limits_{{\begin{array}{*{20}l} {j = 1} \hfill \\ {j \ne i} \hfill \\ {j \in e_{i} } \hfill \\ {e_{j} \ne e_{i} } \hfill \\ \end{array} }}^{{r_{i} }} {\sum\limits_{{\begin{array}{*{20}l} {k = 1} \hfill \\ {k \ne j \ne i} \hfill \\ {k \in e_{j} } \hfill \\ {e_{k} \ne e_{j} \ne e_{i} } \hfill \\ \end{array} }}^{{r_{j} }} {\sum\limits_{q = 1}^{{r_{k} }} {d_{H} (j) \times d_{H} (k) \times JD_{{H_{3} }} (iq)} } } } \right\} $$

15 $$ N_{{2{\text{-}}hyper{\text{-}}path}} = d_{H} (i)\left\{ {\sum\limits_{\begin{gathered} j = 1 \hfill \\ j \ne i \hfill \\ j \in e_{i} \hfill \\ e_{j} \ne e_{i} \hfill \\ \end{gathered} }^{{r_{i} }} {\sum\limits_{\begin{gathered} k = 1 \hfill \\ k \ne j \ne i \hfill \\ k \in e_{j} \hfill \\ e_{k} \ne e_{j} \ne e_{i} \hfill \\ \end{gathered} }^{{r_{j} }} {d_{H} (j) \times d_{H} (k)} } } \right\} $$

where *r*_*i*_ denotes the total number of nodes in the hyper-edge of contains the node *i*, *r*_*j*_ denotes the total number of nodes in the hyper-edge of contains the node *j*; *r*_*k*_ denotes the total number of nodes in the hyper-edge of contains the node *k*; $JD_{{H_{{_{3} }} }} (iq)$ denotes the joint degree of node *i* and node *q*; *e*_*i*_ denotes the hyper-edge containing the node *i*; *e*_*j*_ denotes the hyper-edge containing the node *j*; and *e*_*k*_ denotes the hyper-edge containing the node *k*.

From equations (14) and (15), the number of hyper-triangles of a node is not only related to the hyper-degree of that node but also to the joint degree of that node. Since both the valuation equation of the hyper-clustering coefficient and the expression of the generalized Laplacian matrix of the hyper-network are related to the joint degree of the nodes. For this reason, this paper further explores the relationship between the synchronization ability and the hyper-clustering coefficient of 3-uniform hyper-networks.

## Results

To investigate the influence of the structure of the hyper-networks on their synchronization ability, this paper compares and analyzes the synchronization ability of three types of *k*-uniform ER random hyper-network, *k*-uniform NW small-world hyper-network, and *k*-uniform BA scale-free hyper-network, respectively. The relationship between the synchronization ability and the hyper-clustering coefficient of three types of 3*k*-uniform hyper-networks under different hyper-network structures is also explored. To ensure the validity and reliability of the experimental results, all results in this paper are taken as an average of 50 realization.

### Synchronization ability and hyper-clustering coefficient analyze of *k*-uniform hyper-network

**Figure 2:**
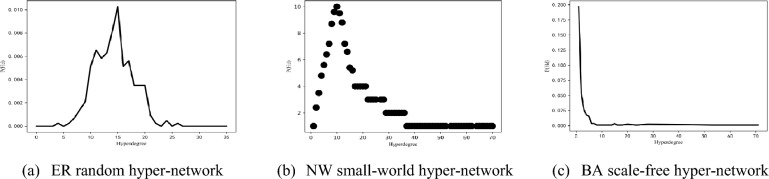
Hyper-degree distribution.

Different structures of hyper-networks exhibit different topological properties, among which the most representative topological property is the hyper-degree distribution of hyper-networks. Under different hyper-network structures, the hyper-degree distribution shows different distribution characteristics, for example, the hyper-degree distribution of ER random hyper-network obeys Poisson distribution; the hyper-degree distribution of NW small-world hyper-network obeys skewed Poisson distribution, while the hyper-degree distribution of BA scale-free hyper-network obeys power-law distribution. Figure 2 shows the hyper-degree distributions of three different types of uniform hyper-networks.

Let *He* is the number of hyper-edges added at each time step during model construction of the BA scale-free hyper-network, *P-NW* is the probability of adding hyper-edges in the NW small-world hyper-network and *P-ER* is the probability of connection hyper-edges in the ER random hyper-network, *N* is the number of node of the hyper-network. When analyzing the synchronization ability of three types of k-uniform hyper-networks, this paper analyzes the effects of node size *N*, *P-ER, P-NW* and *He* on the synchronization ability of three types of k-uniform hyper-networks, and analyzes the hyper- clustering coefficient (*H*_*C*_), respectively.

**Figure 3:**
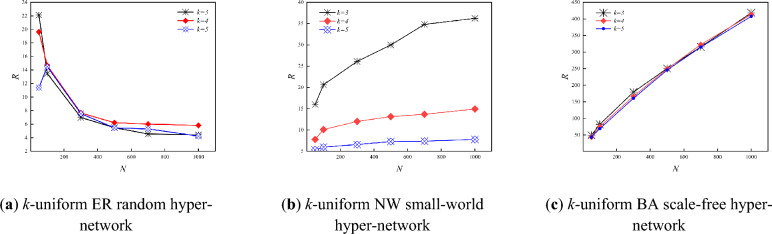
Variation curve of *R* with *N.*

Figure 3 shows the variation of the *R* with *N* for three types of *k*-uniform hyper-networks when *P-ER, P-NW* and *He* is certain (*k* = 3,4,5). From Fig. 3, it can be seen that the *R* of the *k*-uniform ER random hyper-network keep decreasing and those of the *k*-uniform NW small-world hyper-network and the *k*-uniform BA scale-free hyper-network keep increasing as the *N* keeps increasing. For instance when *N* = 300, *R* = 6.9860 for the 3-uniform ER random hyper-network, *R* = 26.104 for the 3-uniform NW small-world hyper-network, and *R* = 179.442 for the 3-uniform BA scale-free hyper-network; when *N* = 1000, *R* = 4.413 for the 3-uniform ER random hyper-network, *R* = 36.235 for the 3-uniform NW small-world hyper-network, and *R* = 418.237 for the 3-uniform BA scale-free hyper-network.

The results in Fig. 3 show that the synchronization ability of the *k*-uniform ER random hyper-network increases with the increasing *N* when the connection hyper-edges probability *P-ER* is certain. The analysis shows that the larger the *N* of the *k*-uniform ER random hyper-network is, the more the number of nodes with the same hyper-degree will be when the probability *P-ER* of the hyper-network is determined. The more homogeneous the *k*-uniform ER random hyper-network is at this point, the easier it is to achieve synchronization. While the synchronization ability of the *k*-uniform NW small-world hyper-network and the *k*-uniform BA scale-free hyper-network shows a decreasing trend with increasing *N*. The analysis shows that during the model construction of the NW small-world hyper-network since the hyper-edge is added to the nearest-neighbor coupling hyper-network with probability *P-NW* and the *k* nodes in the hyper-edges are chosen randomly, this leads to the heterogeneous characteristics of the constructed *k*-uniform NW small-world hyper-network, and the heterogeneity of the *k*-uniform NW small-world hyper-network becomes stronger and the synchronization ability of the hyper-network becomes weaker as the *N* keeps increasing. During the construction of the *k*-uniform BA scale-free hyper-network model, as the size of the hyper-network nodes increases, the stronger the heterogeneity of the *k*-uniform BA scale-free hyper-network exhibits, and the less easy it is to achieve synchronization in the *k*-uniform BA scale-free hyper-network at this time.

**Figure 4:**
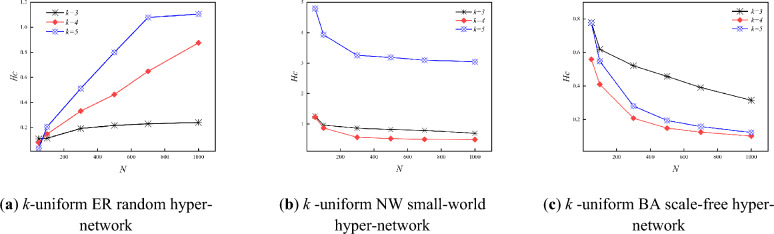
Variation curve of *H*_*C*_ with *N.*

Figure 4 shows the variation of the hyper-clustering coefficient (*H*_*C*_) with *N* for three types of *k*-uniform hyper-network when *P-ER, P-NW* and *He* are certain (*k* = 3,4,5). As can be seen from Fig. 4, the hyper-clustering coefficient of the *k*-uniform ER random hyper-network keeps increasing with the increasing *N*, while the hyper-clustering coefficients of the *k*-uniform NW small-world hyper-network and the *k*-uniform BA scale-free hyper-network keep decreasing. For instance, when *N* = 300, *H*_*C*_ = 0.1922 for the 3-uniform ER random hyper-network, *H*_*C*_ = 0.8610 for the 3-uniform NW small-world hyper-network, *H*_*C*_ = 0.5210 for the 3-uniform BA scale-free hyper-network; when *N* = 1000, *H*_*C*_ = 0.2397 for the 3-uniform ER random hyper-network, *H*_*C*_ =  0.6904 for the 3-uniform NW small-world hyper-network, and *H*_*C*_ = 0.3148 for the 3-uniform BA scale-free hyper-network.

**Figure 5:**
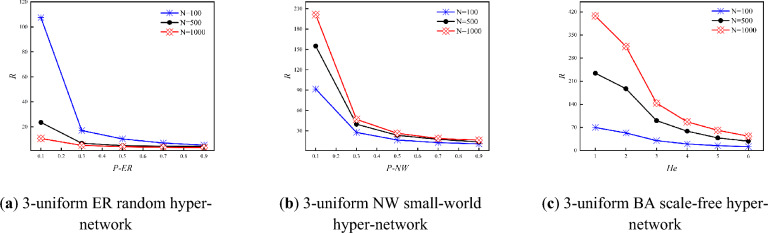
Variation of *R* with *P-ER, P-NW*, *He.*

Figure 5 shows the variation of the synchronization ability of three types of 3-uniform hyper-networks with *P-ER, P-NW* and *He* when the *N* is certain (*N* = 500, 1000). As can be seen from Fig. 5, the *R* of both the 3-uniform ER random hyper-network and the 3-uniform NW small-world hyper-network keep decreasing as *P-ER, P-NW* keeps increasing when the *N* is certain. Meanwhile, when the *N* is certain, the *R* of the 3-uniform BA scale-free hyper-network keeps decreasing as *He* keeps increasing, and the detailed change values are shown in Table 2. While He = *m* for adding *m* hyper-edges at each time step during model construction of the BA scale-free hyper-network.

**Table 2:** Size of the values of the three types of 3-uniform hyper networks *R* with different *P-ER, P-NW* and *He* (*N* = 500).

	*R*				
	*P-ER* = *P-NW* = 0.1 (*He* = 1)	*P-ER* = *P-NW* = 0.2 (*He* = 2)	*P-ER* = *P-NW* = 0.3 (*He* = 3)	*P-ER* = *P-NW* = 0.4 (*He* = 4)	*P-ER* = *P-NW* = 0.5 (*He* = 5)
ER_hyper-network	23.523	6.869	4.902	4.471	4.373
NW_hyper-network	154.983	39.837	23.422	17.459	13.696
BA_hyper-network	234.1567	187.2823	90.6349	58.723	38.2426

Combining Table 2 and Fig. 5, it can be seen that when the *N* is certain, the synchronization ability of the 3-uniform ER random hyper-network keeps increasing with the increasing probability *P-ER*. The analysis shows that when the *N* is certain, the larger the connection hyper-edge probability *P-ER*, the more the number of hyper-edges in the hyper-network, the closer the connection between nodes, and the easier it is to achieve synchronization in the 3-uniform ER random hyper-network at this time. At the same time, the synchronization ability of the 3-uniform NW small-world hyper-network is increasing with the increasing probability *P-NW*. The analysis shows that when the *N* is certain, the larger the probability *P-NW*, the greater the number of hyper-edges joined in the hyper-network, the more dense the connections between nodes in the hyper-network, and the easier it is to achieve synchronization in the 3-uniform NW small-world hyper-network at this time. The synchronization ability of the 3-uniform BA scale-free hyper-network increases with the increasing number of hyper-edges *He* added at each time. The analysis shows that during the construction of the 3-uniform BA scale-free hyper-network model, one node and *He* hyper-edges are added at each time step, and the more hyper-edges are added, the node will establish connections with more nodes, and the easier the 3-uniform BA scale-free hyper-network is to achieve synchronization at this time.

**Figure 6:**
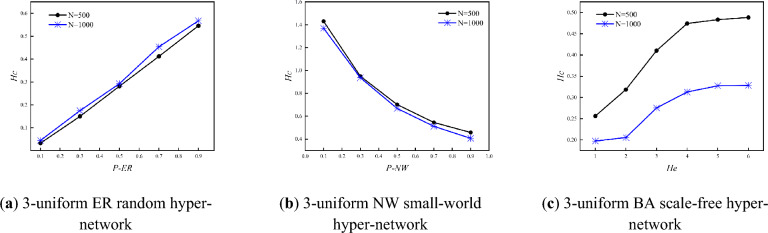
Variation of *H*_*C*_ with *P-ER, P-NW, He.*

Figure 6 shows the variation of the hyper-clustering coefficient with *P-ER, P-NW* and *He* for three types of 3-uniform hyper-networks when the *N* is certain (*N* = 500,1000). The results in Fig. 6 show that the hyper-clustering coefficient of the 3-uniform ER random hyper-network increases continuously with the increasing probability *P-ER*; the hyper-clustering coefficient of the 3-uniform NW small-world hyper-network decreases continuously with the increasing probability *P-NW*; and the hyper- clustering coefficient of the 3-uniform BA scale-free hyper-network increases continuously with the increasing *He*.

**Figure 7:**
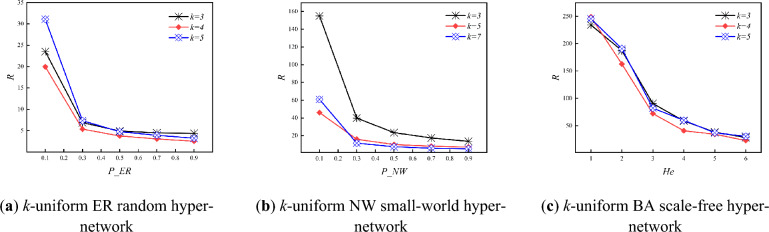
Variation of *R* with *P_ER, P_NW* and *He* when *k* = 3,4,5

Figure 7 shows the variation of synchronization ability with *P_ER, P_NW* and *He* for three types of *k*-uniform hyper-networks when *k* is different. From Fig. 7, it can be seen that in three types of *k*-uniform hyper-networks, the eigenvalue ratio under different uniformities keeps decreasing as the *P_ER, P_NW* and *He* keeps increasing at *k* = 3,4,5, when the synchronization ability of three types of *k*-uniform hyper-networks keeps increasing. Specific analysis is the same as that of the 3-uniform hyper-network.

**Figure 8:**
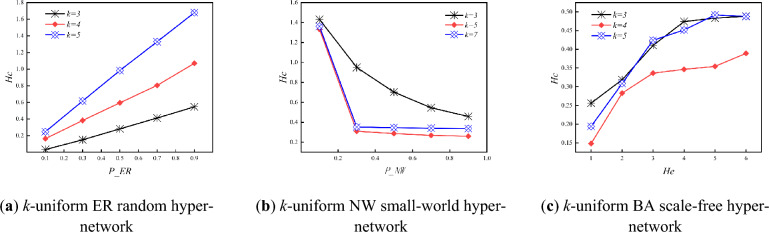
Variation of *H*_*C*_ with *P_ER, P_NW* and *He* when *k* is different.

Figure 8 shows the variation of hyper-clustering coefficient with *P_ER, P_NW* and *He* for three types of *k*-uniform hyper-networks when *k* is different. From Fig. 8, it can be seen that as the *P_ER* and *He* keeps increasing, the hyper-clustering coefficient of *k*-uniform ER random hyper-networks and *k*-uniform BA scale free hyper-networks continues to increase; while the hyper-clustering coefficients of *k*-uniform NW small-world hyper-network continue to decrease under different uniformities at *k* = 3,5,7. Specific analysis is the same as that of the 3-uniform hyper-network.

### Relationship between the synchronization ability and hyper-clustering coefficient analysis of the *k*-uniform hyper-network

In analyzing the relationship between synchronization ability and hyper-clustering coefficients of three types of *k*-uniform hyper-networks, this paper explores the different relationships between synchronization ability and hyper-clustering coefficient of *k*-uniform hyper-networks with different *N*, *P-ER, P-NW* and *He*, respectively.

**Figure 9:**
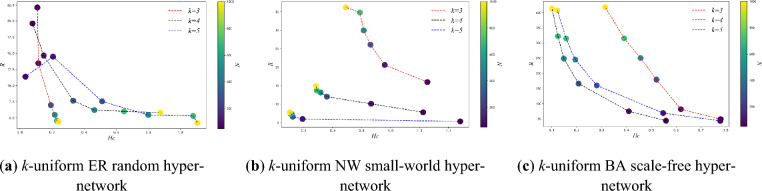
Relationship between *R* and *H*_*C*_ when *N* is different (*k* = 3,4,5).

Figure 9 shows the variation of the *R* and the hyper-clustering coefficient *H*_*C*_ for the three types of *k*-uniform hyper-networks with different *N*(*k* = 3,4,5). From the simulation data given in Table 3, it can be seen that in the *k*-uniform ER random hyper-network, the hyper-clustering coefficient increases and the *R* decreases as the *N* continues to increase. In the *k*-uniform NW small-world hyper-network, the hyper-clustering coefficient keeps decreasing and the *R* keeps increasing as the *N* keeps increasing; in the *k*-uniform BA scale-free hyper-network, the hyper- clustering coefficient keeps decreasing and the *R* keeps increasing as the *N* keeps increasing. As can be seen from Fig. 9, the *k*-uniform NW small-world hyper-networks and the *k*-uniform BA scale-free hyper-networks show the same changing trend, i.e., the hyper- clustering coefficient decreasing and the *R* increases as the *N* keeps increasing. And the *k*-uniform ER random hyper-network exhibits a different trend from the other two types of *k*-uniform hyper-networks, i.e., the hyper-clustering coefficient keeps increasing and the *R* keeps decreasing as the *N* keeps increasing.

**Table 3:** Numerical magnitudes of *R* and *H*_*C*_ for the three types of *k*-uniform hyper-networks at different *N.*

		*N* = 100	*N* = 500	*N* = 700	*N* = 1000
ER_hyper-network					
*k* = 3	*R*	13.482	5.477	4.5463	4.413
	*H*_*C*_	0.1143	0.2167	0.2296	0.2398
*k* = 4	*R*	14.6651	6.2192	6.0182	5.8242
	*H*_*C*_	0.1482	0.4637	0.6483	0.8756
*k* = 5	*R*	14.4849	5.4539	5.3025	4.2277
	*H*_*C*_	0.2056	0.7998	1.0787	1.1058
NW_hyper-network					
*k* = 3	*R*	20.625	29.971	34.814	36.235
	*H*_*C*_	0.9593	0.8144	0.7878	0.6904
*k* = 5	*R*	10.0984	13.142	13.6896	14.9198
	*H*_*C*_	0.5603	0.517	0.4921	0.4855
*k* = 7	*R*	5.9754	7.2791	7.3702	7.7857
	*H*_*C*_	0.9931	0.6186	0.5095	0.5056
BA_hyper-network					
*k* = 3	*R*	81.999	250.642	315.508	418.237
	*H*_*C*_	0.619	0.457	0.391	0.3148
*k* = 4	*R*	75.1547	248.8911	322.4018	413.3311
	*H*_*C*_	0.4098	0.1482	0.1245	0.1007
*k* = 5	*R*	69.1208	245.5323	314.8336	407.5843
	*H*_*C*_	0.5477	0.1942	0.1575	0.1224

From Fig. 9, it can be seen that although the three types of *k*-uniform hyper-networks show different trends between the *R* and hyper-clustering coefficient, all three types of *k*-uniform hyper-networks exhibit smaller *R* for larger hyper-clustering coefficients, i.e., the greater the hyper-clustering coefficient, the greater the synchronization ability. When considering the effect of a single variable on its synchronization ability under different hyper-network structures, the trend of the hyper-clustering coefficient can be used to characterize the trend of the synchronization ability of a uniform hyper-network.

**Figure 10:**
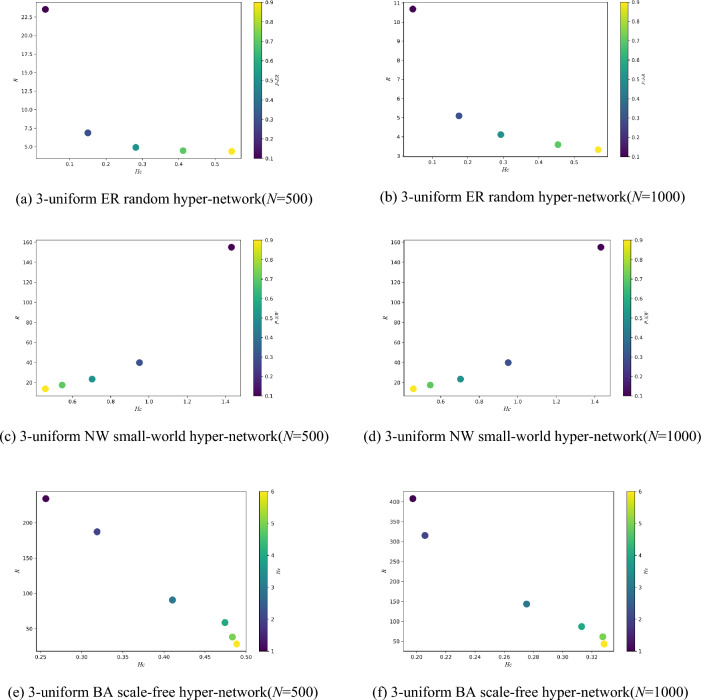
Relationship between *R* and *H*_*C*_ when *P-ER, P-NW, He* are different.

Figure 10 shows the different relationships between the synchronization ability and the hyper-clustering coefficient for the three types of 3-uniform hyper-networks with different *P-ER, P-NW* and *He*. From the data in Table 4, it can be seen that the hyper-clustering coefficient of the 3-uniform ER random hyper-network keeps increasing and the *R* keeps decreasing as the probability of connection hyper-edges *P-ER* keeps increasing; the hyper-clustering coefficient of the 3-uniform NW small-world hyper-network keeps decreasing and the *R* keeps decreasing as the probability of adding hyper-edges *P-NW* keeps increasing; the hyper- clustering coefficient of the 3-uniform BA scale-free hyper-network keeps increasing and the *R* keeps decreasing as *He* keeps increasing. As can be seen from Fig. 10, among the three types of 3-uniform hyper-networks, the 3-uniform ER random hyper-network and the 3-uniform BA scale-free hyper-network show the same changing trend, i.e., the hyper-clustering coefficient keeps increasing and the *R* keeps decreasing with the increasing probability of connection hyper-edges *P*-ER and *He*. In contrast, the 3-uniform NW small-world hyper-network indicates a different trend from the other two types, i.e., the hyper-clustering coefficient keeps decreasing and the *R* keeps decreasing with the increasing probability of adding hyper-edges *P-NW*.

**Table 4:** Numerical magnitudes of *R* and *H*_*C*_ for three types of *k*-uniform hyper-networks for different *P-ER, P-NW* and *He.*

		*P-ER* = *0.1*	*P-ER* = 0.3	*P-ER* = 0.5	*P-ER* = 0.7	*P-ER* = 0.9	
ER_hyper-network							
*k* = 3	*R*	23.523	6.869	4.902	4.471	4.373	
	*H*_*C*_	0.0332	0.1501	0.282	0.4122	0.546	
*k* = 4	*R*	19.9581	5.344	3.758	3.0376	2.5302	
	*H*_*C*_	0.1639	0.3826	0.5952	0.8043	1.0702	
*k* = 5	*R*	31.079	7.3227	4.751	3.9171	3.2209	
	*H*_*C*_	0.2474	0.617	0.9834	1.3294	1.6786	

		*k* = *3*	*P-NW* = 0.3	*P-NW* = 0.5	*P-NW* = 0.7	*P-NW* = 0.9	
NW_hyper-network							
*k* = 3	*R*	154.983	39.837	23.422	17.459	13.696	
	*H*_*C*_	1.4312	0.9503	0.7026	0.5456	0.4581	
*k* = 5	*R*	45.9799	16.0349	10.014	8.1558	7.1328	
	*H*_*C*_	1.3324	0.3099	0.2871	0.2689	0.2599	
*k* = 7	*R*	60.8713	11.5462	7.5317	5.8475	5.2109	
	*H*_*C*_	1.3614	0.353	0.3458	0.3407	0.3373	

		*He* = 1	*He* = 2	*He* = 3	*He* = 4	*He* = 5	*He* = 6
BA_hyper-network							
*k* = 3	*R*	234.157	187.2823	90.6349	58.723	38.2426	28.438
	*H*_*C*_	0.2563	0.3187	0.4104	0.4743	0.4833	0.4885
*k* = 4	*R*	248.8911	162.7441	72.1714	40.7534	34.5272	22.9592
	*H*_*C*_	0.1482	0.2829	0.3364	0.3465	0.3544	0.3893
*k* = 5	*R*	245.5323	190.7416	82.1763	59.8465	37.1564	30.5083
	*H*_*C*_	0.1942	0.3085	0.4241	0.4519	0.492	0.5079

From Fig. 10, it can be seen that among the three types of 3-uniform hyper-networks, with the increasing of *P-ER* and *He*, both 3-uniform ER random hyper-networks and 3-uniform BA scale-free hyper-networks exhibit the larger the probability of connection hyper-edges *P-ER* and *He*, the larger the hyper- clustering coefficient and the smaller the *R*, i.e., with the increase of *P-ER* and *He*, the larger the hyper- clustering coefficient and the stronger the synchronization ability. And the 3-uniform NW small-world hyper-networks show a different trend from the other two types of uniform hyper-networks, i.e., the larger the probability of adding hyper-edges *P-NW*, the smaller the hyper-clustering coefficient, the smaller the *R*, and the stronger the synchronization ability. It is thus clear that under different hyper-network structures and considering different variables, although the synchronization ability of three types of uniform hyper-networks increases with the increase of different variables, the hyper-clustering coefficient of the three types of uniform hyper-networks shows different trends with the increase of different variables. When both the structure and the variables of the considered hyper-networks are different, it is not possible to characterize the trend of the hyper-network synchronization ability uniformly using the trend of the hyper-clustering coefficient. Yet, when the hyper-network structure is determined, if only a single variable is considered, the trend of the change of the hyper-clustering coefficient can be used to characterize the trend of the hyper-network synchronization ability now. For instance, when considering the variation of the synchronization ability of a 3-uniform ER random hyper-network with the probability of connection hyper-edges *P-ER*, the trend of the hyper-clustering coefficient can be used to characterize the trend of the synchronization ability.

**Figure 11:**
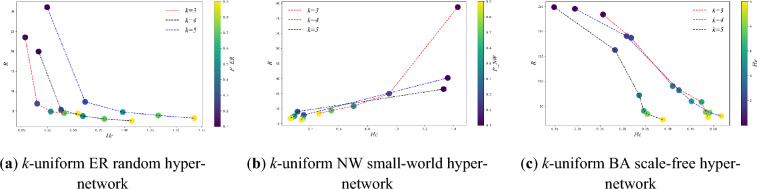
Relationship between *R* and *H*_*C*_ when *P-ER, P-NW, He* are different (*k* = 3,4,5).

From Figure 11, it can be seen that among the three types of *k*-uniform hyper-networks, with the increasing of *P-ER* and *He*, both *k*-uniform ER random hyper-networks and k-uniform BA scale-free hyper-networks exhibit the larger the probability of connection hyper-edges *P-ER* and *He*, the larger the hyper- clustering coefficient and the smaller the *R*, i.e., with the increase of *P-ER* and *He*, the larger the hyper- clustering coefficient and the stronger the synchronization ability. And the *k*-uniform NW small-world hyper-networks show a different trend from the other two types of uniform hyper-networks, i.e., the larger the probability of adding hyper-edges *P-NW*, the smaller the hyper-clustering coefficient, the smaller the *R*, and the stronger the synchronization ability. Specific analysis is the same as that of the 3-uniform hyper-network.

### Synchronization ability comparison of *k*-uniform hyper-network

To obtain the strength of synchronization ability of three types of *k*-uniform hyper-networks. This paper compares the size of the eigenvalue ratio *R* of three types of 3-uniform hyper-networks with the same node size *N*, then obtains the strength of synchronization ability of three types of 3-uniform hyper-networks.

**Figure 12:**
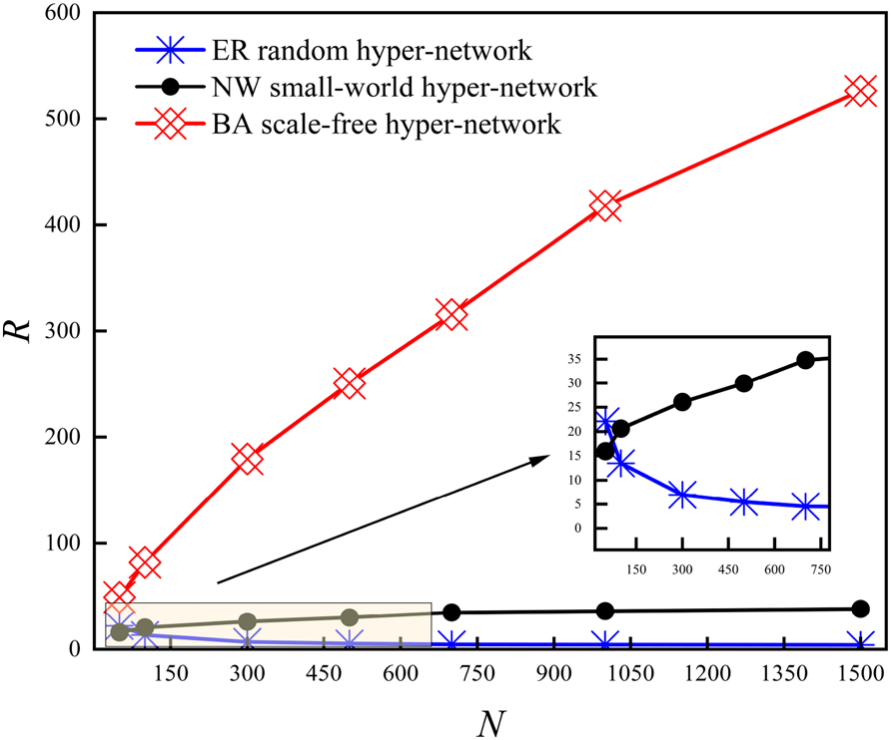
Comparison of three types of uniform hyper-networks *R* with different *N.*

Figure 12 shows the variation of the *R* for the three types of 3-uniform hyper-networks as the *N* varies. It can be seen that when the *N* is fixed, the one with the largest *R* among the three types of 3-uniform hyper-networks is the 3-uniform BA scale-free hyper-network, followed by the 3-uniform NW small-world hyper-network, and the smallest is the 3-uniform ER random hyper-network, i.e., the hyper-network with the best synchronization ability among the three types of 3-uniform hyper-networks is the 3-uniform ER random hyper-network, followed by the 3-uniform NW small-world hyper-network, and finally the 3-uniform BA scale-free hyper-network. As can be seen from Fig. 12, with the increasing *N*, only the *R* curves of the 3-uniform ER random hyper-network show a decreasing trend, while the *R* curves of the 3-uniform NW small-world hyper-network and the 3-uniform BA scale-free hyper-network show an increasing trend. The trend of the *R* curves of the three types of 3-uniform hyper-networks shows that the best synchronization ability is 3-uniform ER random hyper-networks when the *N* is large, which is also consistent with the results of the comparison of the synchronization ability of several types of networks in complex networks.

**Figure 13:**
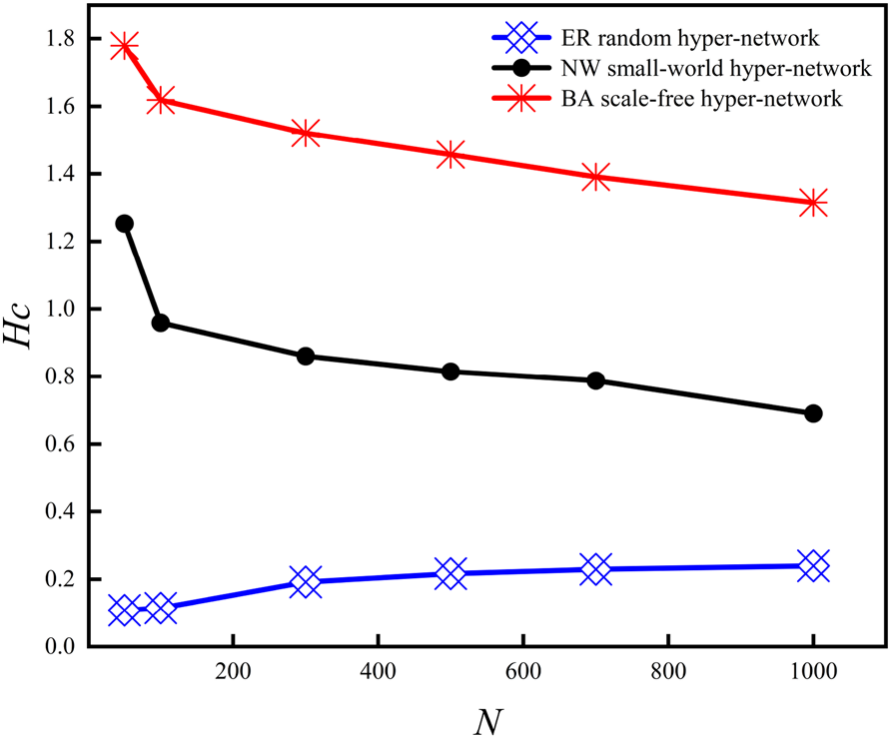
Comparison of three types of uniform hyper-networks *H*_*C*_ with different *N.*

Figure 13 shows the variation of the hyper-clustering coefficient of the three types of 3-uniform hyper-networks as the *N* changes. The results in Fig. 13 show that the hyper-clustering coefficient curves of the 3-uniform ER random hyper-network show an increasing trend with the increasing *N*, while the hyper-clustering coefficient curves of the 3-uniform NW small-world hyper-networks and the 3-uniform BA scale-free hyper-networks show a decreasing trend.

Table 3 shows the relationship between the *R* and the hyper-clustering coefficient *H*_*C*_ for three types of k-uniform hyper-networks with different *N*(*k* = 3,4,5). The results shown in Table 3 indicate that the *R* of the k-uniform NW small-world hyper-network and the *k*-uniform BA scale-free hyper-network decrease with increasing hyper-clustering coefficient as the *N* continues to increase, i.e., the smaller the hyper- clustering coefficient in the *k*-uniform NW small-world hyper-network and the *k*-uniform BA scale-free hyper-network, the stronger the synchronization ability of the hyper-network. On the contrary, in the *k*-uniform ER random hyper-network, the *R* increases with the decreasing hyper-clustering coefficient as the *N* keeps increasing, i.e., the smaller the hyper-clustering coefficient in the *k*-uniform ER random hyper-network, the stronger the synchronization ability of the hyper-network. In summary, with the increasing size of nodes, the trend of synchronization ability of all three types of uniform hyper-networks can be characterized by the trend of the hyper-clustering coefficient.

Table 4 shows the relationship between the *R* and the hyper-clustering coefficient *H*_*C*_ for two types of hyper-networks with different *P-ER, P-NW*(*k* = 3,4,5). From Table 4, it can be seen that in the *k*-uniform ER random hyper-network, when the *N* is certain, the hyper-clustering coefficient increases continuously with the increasing probability of connection hyper-edges *P-ER* and the *R* decreases continuously; while in the *k*-uniform NW small-world hyper-network, when the *N* is certain, the hyper-clustering coefficient decreasing continuously with the increasing probability of adding hyper-edges *P-NW* and the *R* decreases continuously. Under both types of hyper-network structures, the synchronization ability is enhanced with the increasing *P-ER, P-NW*, but the hyper-clustering coefficient of the two types of hyper-networks show different trends with the increasing *P-ER, P-NW* at this time.

In summary, when the *N* of *k*-uniform hyper-networks is certain, the synchronization ability of both *k*-uniform ER random hyper-network and *k*-uniform NW small-world hyper-network increases with the increase of *P-ER, P-NW*, but the hyper- clustering coefficient of the two types of hyper-networks show different trends. Therefore, when considering the effects of two different variables, the probability of connection hyper-edges *P-ER* and the probability of adding hyper-edges *P-NW* on the synchronization ability of the two types of *k*-uniform hyper-networks, the synchronization ability of the two types of hyper-networks shows different trends about the hyper-clustering coefficient. It is thus clear that the variation of synchronization ability of the two types of hyper-networks cannot be measured purely by the indicator of hyper-clustering coefficient when both the structure and variables of the considered hyper-networks are different.

## Discussion

In this paper, we propose a Kuramoto model expression that is more suitable for describing the synchronization of *k*-uniform hyper-networks and propose an expression for the generalized Laplacian matrix of uniform hyper-networks. Based on the size of the *R* as the basis for judging the strength of synchronization ability, we compare and analyze the synchronization ability of three types of *k*-uniform hyper-networks and obtain different relationships between the synchronization ability of hyper-networks and the hyper-clustering coefficient. The strongest synchronization ability among the three types of *k*-uniform hyper-networks obtained by experimental analyze is the *k*-uniform ER random hyper-network, followed by the *k*-uniform NW small-world hyper-network, and the worst synchronization ability is the *k*-uniform BA scale-free hyper-network. In analyzing the effect of *N* on the synchronization ability of the three types of uniform hyper-networks, the *k*-uniform ER random hyper-network exhibits increasing synchronization ability with increasing *N*, while the *k*-uniform NW small-world hyper-network and the *k*-uniform BA scale-free hyper-network exhibit decreasing synchronization ability with increasing *N*. In analyzing the effects of parameters on the synchronization ability of uniform hyper-networks, the *k*-uniform ER random hyper-network exhibits increasing synchronization ability with increasing probability of connection hyper-edges *P-ER*. The *k*-uniform NW small-world hyper-network exhibits increasing synchronization ability with increasing probability of adding hyper-edges *P-NW*. The *k*-uniform BA scale-free hyper-network exhibits increasing synchronization ability with increasing number of joining hyper-edges *He* per time step. In this paper, the relationship between the hyper-clustering coefficient and the synchronization ability is also explored while analyzing the strength of the synchronization ability of uniform hyper-networks. It is concluded that when the influence of a single variable on the synchronization ability is considered under different hyper-network structures, the changing trend of the synchronization ability can be characterized by the changing trend of the hyper-clustering coefficient, but when both the considered hyper-network structures and variables are different, the changing trend of the hyper-clustering coefficient and the changing trend of the synchronization ability is different under different hyper-network structures, and the change of the synchronization ability of *k*-uniform hyper-networks cannot be measured by a single indicator of the hyper-clustering coefficient at this time.

## Data Availability

The processed data required to reproduce these findings cannot be shared at this time as the data also forms part of an ongoing study. In future, the processed data that support the findings of this study are available from the corresponding author upon reasonable request.
