# Author Correction: Synchronization analyze of *k*-uniform hyper-networks

**DOI:** 10.1038/s41598-024-65403-8

**Published:** 2024-06-24

**Authors:** Juan Du, Xiujuan Ma, Fuxiang Ma, Wenqian Yu

**Affiliations:** 1 School of Computer Science, Qinghai Normal University, Xining, 810000 Qinghai China; 2 The State Key Laboratory of Tibetan Intelligent Information Processing and Application, Qinghai Normal University, Xining, 810016 Qinghai China

Correction to: *Scientific Reports* 10.1038/s41598-024-56198-9, published online 13 March 2024

The original version of this Article contained errors in the Acknowledgements section.

“The authors acknowledge the support of the Construction Project for Innovation Platform of Qinghai Province (No. 2022ZJT02), National Natural Science Foundation of China (No. 61603206), The State Key Laboratory of Tibetan Intelligent Information Processing and Application (2020-ZJ-Y05). We are grateful to the anonymous referees for their helpful comments.”

now reads:

“The authors acknowledge the support of the Natural Science Foundation of Qinghai Province in China (NO. 2019-ZJ-7012), Construction Project for Innovation Platform of Qinghai Province (No. 2022ZJT02), and National Natural Science Foundation of China (No. 61603206). We are grateful to the anonymous referees for their helpful comments.”

The original Article has been corrected.

